# Protocells Models in Origin of Life and Synthetic Biology

**DOI:** 10.3390/life5041700

**Published:** 2015-12-08

**Authors:** Pasquale Stano, Fabio Mavelli

**Affiliations:** 1 Sciences Department, Roma Tre University; Viale G. Marconi 446, I-00146 Rome, Italy; 2 Chemistry Department, University of Bari; Via E. Orabona 4, I-70125 Bari, Italy

Over recent years, the investigation of protocells (here intended either as primitive cell models, either as synthetic cell-like systems of minimal complexity) has gained prominence in an interdisciplinary field embracing both origins-of-life studies [1,2,3,4] and modern synthetic biology [5,6,7,8,9,10]. Protocells have stimulated, and continue to stimulate, the ingenuity of an ever-growing number of scientists, whose contributions will be determinant of the progress and success of the field.

Protocell research is aimed at designing, constructing, and characterizing micro-compartmentalized structures that share with primitive cells or with modern living cells their peculiar static and dynamic organization. The Ganti’s *chemoton* [11] or the Maturana-Varela *autopoietic systems* [12] have often been taken as theoretical framework for such enterprise.

**Figure 1:**
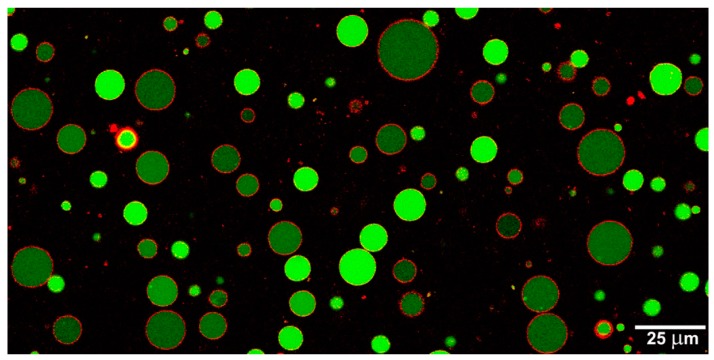
Giant lipid vesicles [25] are often used for constructing cell-like systems. The picture shows two populations of calcein-filled giant vesicles made by lecithin, whose membranes have been stained by trypan blue. Enzymes and other macromolecules are easily encapsulated inside giant vesicles, allowing the construction of micro-compartmentalized systems capable of programmable behavior.

From an experimental point of view, several approaches are currently under scrutiny. Most of them rely on vesicles (see Figure 1) (both from fatty acids or phospholipids), but polymer vesicles [13] and coacervates have been also used [14]. The choice of the molecular entities for constructing protocells embraces strictly prebiotic, semi-synthetic, or fully synthetic species, although hybridization among these approaches is often present. It is important to remark, however, that, despite the apparent diversity in the chemical details, all recent efforts and discoveries have collectively improved the knowledge of the physico-chemical and organizational conditions that shaped and possibly promoted the transition from non-living to living matter [15]. This kind of new knowledge—generated through a “constructive paradigm” [16,17]—helps shed light on the origin of early cells on earth, and at the same time it will enable novel *synthetic cell* technologies that might be useful for applicative purposes. It is very peculiar that research on protocells in origin of life scenarios intersects with the most advanced trends in synthetic biology [18], and that the laboratory assembly of artificial cellular structures embraces (bio)physics, (bio)chemistry, (bio)engineering and other specialties, last but not least *in silico* approaches.

Indeed, one important advantage of protocell systems is that their reduced complexity (when compared to living systems) allows detailed studies by computational methods, according to both deterministic and stochastic approaches [19,20,21,22,23,24]. The construction of minimal cell-like systems allows for the first time a direct comparison between experiments and numerical models in molecular systems of manageable complexity, where the exquisitely intrinsic stochastic chemical reactivity is coupled with membrane transport, micro-compartmentation of multiple solutes and the consequent extrinsic stochastic phenomena. The latter, ultimately, mark as a signature the basic nature of small compartments and their individuality (with the resulting issues of selection among similar but non identical micro-systems). Numerical methods facilitate the discoveries of genuine effects that cannot be pinpointed *a priori,* and that can actually be confirmed experimentally. Computational studies, especially when based on parameters inferred by experimental data, allow for the exploration of hypothetical dynamical behaviors that are difficult to investigate experimentally. All together, these issues call for a systematic use of simulations flanking the wet laboratory (or, in short, the need for an *in silico vesicle lab*).

Altogether, *in silico* and *in vitro* investigations are paving the way to a novel research arena that appears to be both very rich (thanks to its intrinsic interdisciplinary character) and promising (because only via synthetic/constructive approaches is it possible to enquire about the features of simple, early cells). This approach also stimulates more theoretical considerations with respect to intriguing questions, such as “what is life?” and further supports abiogenesis as the theoretical framework for understanding the emergence of living systems on Earth.

Minimal cell models are embodiments of system theory applied to living organisms. This is now an exciting multidisciplinary research area mainly aimed at identifying the physico-chemical constraints (or unexpected and helpful emerging features) that are pertinent to the organization of dynamic chemical networks in the form of micro-compartments. More generally, it deals with the chemistry of systems out-of-equilibrium or in stationary dynamical states (see the concept of *systems chemistry* [26]). Thanks to the efforts of many authors, which we greatly acknowledge, this Special Issue aims at placing protocells research under the spotlight as one exciting scientific challenge for current and future scientists.
